# Smart Polymeric Delivery System for Antitumor and Antimicrobial Photodynamic Therapy

**DOI:** 10.3389/fbioe.2021.783354

**Published:** 2021-11-04

**Authors:** Zhijia Wang, Fu-Jian Xu, Bingran Yu

**Affiliations:** Laboratory of Biomedical Materials and Key Lab of Biomedical Materials of Natural Macromolecules Beijing University of Chemical Technology, Ministry of Education, Beijing University of Chemical Technology, Beijing, China

**Keywords:** polymer, target, activatable, photodynamic therapy, antibacterial

## Abstract

Photodynamic therapy (PDT) has attracted tremendous attention in the antitumor and antimicrobial areas. To enhance the water solubility of photosensitizers and facilitate their accumulation in the tumor/infection site, polymeric materials are frequently explored as delivery systems, which are expected to show target and controllable activation of photosensitizers. This review introduces the smart polymeric delivery systems for the PDT of tumor and bacterial infections. In particular, strategies that are tumor/bacteria targeted or activatable by the tumor/bacteria microenvironment such as enzyme/pH/reactive oxygen species (ROS) are summarized. The similarities and differences of polymeric delivery systems in antitumor and antimicrobial PDT are compared. Finally, the potential challenges and perspectives of those polymeric delivery systems are discussed.

## Introduction

Photodynamic therapy (PDT) has attracted intensive attention for the treatment of tumor and bacteria during recent years(Celli et al., 2010; Bugaj, 2011; Kamkaew et al., 2013; Lincoln et al., 2013; Tian et al., 2013). For instance, for traditional cancer therapy strategies such as chemotherapy and radiotherapy, patients often suffer from severe side effects, limited tumor suppression, and unavoidable regrowth of metastatic tumor. In comparison, PDT has high spatiotemporal selectivity in triggering tumor cell death and can generate immune response (Wang et al., 2020), showing advantages over the traditional cancer therapies. On the other hand, concerning anti-bacterial therapy, antibiotic resistance problems become increasingly prominent (Maisch et al., 2004). PDT is expected to be a promising alternative to antibiotic therapy for combating bacterial infections (Klausen et al., 2020). PDT therapeutics such as Porfimer sodium, ALA, and Verteporfin have been approved worldwide to be used in clinics (Agostinis et al., 2011). PDT has been approved to treat some diseases in clinical trials, such as premalignant tumors, cutaneous malignant tumors, tumors of the head, neck, and oral cavity, lung, gastrointestinal, and other tumors (Brown et al., 2004), viral lesions, acne, gastric infection by *Helicobacter pylori,* and brain abcesses (Hamblin and Hasan, 2004).

**FIGURE 1:**
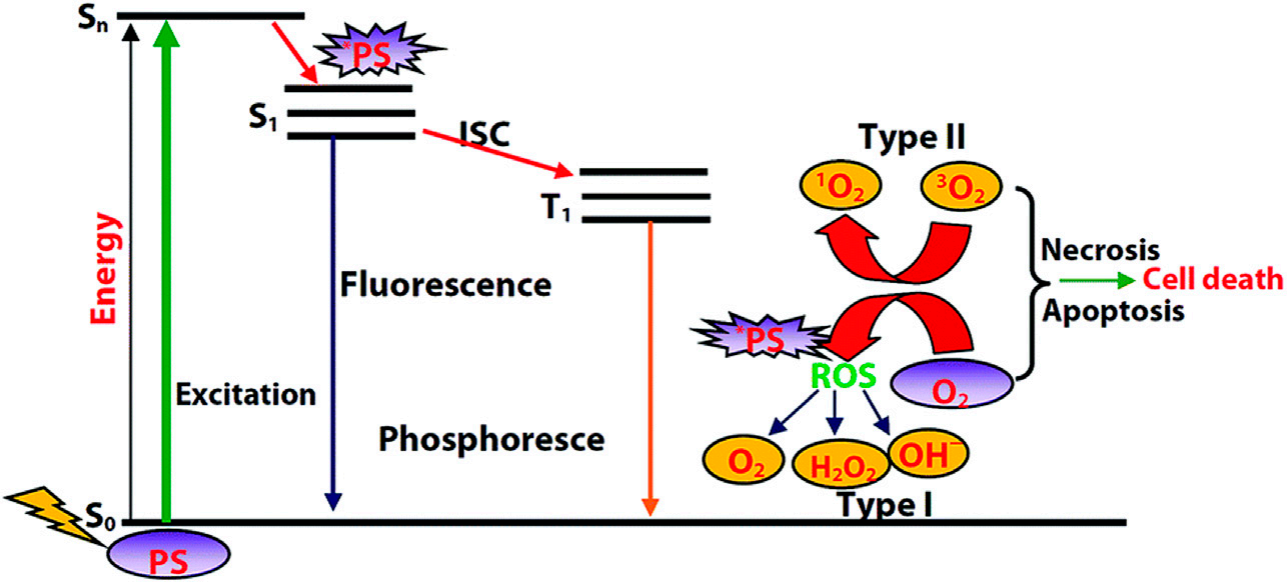
Diagram illustrating the process of PDT. Reprinted with permission from Majumdar et al. (2014).

The mechanism of PDT is described in Figure 1. With light irradiation, the photosensitizer is promoted to its singlet excited state, followed by intersystem crossing to its triplet state. As the transition from triplet excited state (T_1_) to ground state is spin forbidden, this T_1_ state is relatively long-lived and can react with molecular oxygen. Concerning the reaction type between the T_1_ state and molecular oxygen, there are two possibilities. Electron transfer between the T_1_ state and molecular oxygen will initially produce a superoxide anion (O_2_^•−^), and subsequently produce other ROS such as hydroxyl radical or hydrogen peroxide that is toxic to cells or bacteria, which is the so-called Type I PDT. Energy transfer between the T_1_ state and molecular oxygen will produce toxic singlet oxygen (^1^O_2_) to kill tumor cells or bacteria, which is Type II PDT.

It is obvious that triplet photosensitizer, light, and oxygen are three key factors of PDT. Among these, triplet photosensitizer plays a leading role (Majumdar et al., 2014). Although a variety of conventional photosensitizers such as porphyrin derivatives (e.g., chlorin e6, PpIX), phthalocyanine derivatives (e.g., ZnPc), and some newly developed triplet photosensitizers such as Bodipy derivatives have PDT ability (Detty et al., 2004), their water solubility is unsatisfactory. The delivery of those photosensitizers to tumor or infected site is a critical issue.

Polymers consist of many structural units or monomers that are connected with a covalent bond; hence, they have a super high molecular weight. Some polymers play the role of triplet photosensitizers as they can produce ROS (Blacha-Grzechnik, 2021). Some reports found that polymers may have enhanced ROS production ability than monomers, which might be due to the increased number of vibrational energy levels and broader energy bands of polymers as compared with monomers (Wu et al., 2018c; Liu et al., 2021). More commonly, polymers functioned as carries of organic triplet photosensitizer, which increases its water solubility and its accumulation in the tumor or infected site (Regehly et al., 2007; Lee et al., 2009; Park S. Y. et al., 2011; Huang et al., 2016; Cui et al., 2019). In addition, small molecules tend to be cleaned up quickly from the body; hence, the polymers are expected to extend the circulation time. Polymers can also serve as a gene delivery vector, e.g., the delivery of a genetically encoded photosensitizer (such as killerred, miniSOG) for PDT cancer treatment (Sarkisyan et al., 2015; Tseng et al., 2015). Polymers have versatile functions and can combine with other therapy strategies, achieving multimodal therapy (Li and Pu, 2020). For instance, polymers can act as prodrugs for PDT and chemotherapy (Xu et al., 2016; Wang et al., 2019; Lu L. et al., 2020), the combination of polymer and cytotoxic enzymes shows outstanding tumor treatment efficacy (Li et al., 2019c); it can also function as a blockader to intervene in the protein biosynthesis to depress the tumor (Li et al., 2019b); the polymers become stimulants in the combination of PDT and immunotherapy (Li et al., 2019a)

Smart polymers that can target tumor/infected tissues or be activatable by the tumor/bacterial microenvironment are highly desired. Although the PDT has relatively high spatial selectivity, there is still great concern about the skin phototoxicity. For instance, patients treated with porphyrin derivatives have to stay in the dark for at least 72 h, which is a great psychological challenge to patients. An activatable delivery system is beneficial to reduce the skin phototoxicity. Small organic molecules tend to be cleared out from the body, and an activatable polymeric delivery system can protect the small photosensitizer and achieve on site release of photosensitizer. Moreover, the nonspecifically distribution of the triplet photosensitizer in the whole body requires a higher dose of photosensitizer, which is more likely to lead to organ damage; a specific accumulation of the photosensitizer to the lesion site is beneficial to reduce administration dosage, elevate the therapeutic efficacy, and play down the side effects. Hence, developing smart polymeric delivery systems that can target the tumor/bacterial site or activate by the tumor/bacterial microenvironment is urgent. In recent years, elegant works have been conducted in these areas (Hoffman, 2008; Li and Huh, 2014; Kamaly et al., 2016; Chen et al., 2017; Wu L. et al., 2018; Wu et al., 2018b; Li and Yan, 2018; Li et al., 2020; Zheng et al., 2020; Han et al., 2021). This review will give a brief summary on the smart polymeric delivery system for antitumor and antimicrobial photodynamic therapy. Note that due to the limitation of the space, we only selected part of typical articles as examples to introduce in detail. The authors apologize for omitting some important and elegant works in advance.

## Smart Polymeric Delivery System for Antitumor Photodynamic Therapy

Cancer has been a leading cause of death all over the world (Ferlay et al., 2010). Data from the World Health Organization (WHO) shows that nearly 10 million deaths from cancer occurred in 2020. Traditional cancer therapy strategies such as chemotherapy and radiotherapy have severe side effects (Mitsunaga et al., 2011; Wang and Guo, 2013; Lucky et al., 2015) Moreover, there are great concerns on the drug resistance and radioactivity for chemotherapy and radiotherapy, respectively (Mitsunaga et al., 2011; Wang and Guo, 2013; Lucky et al., 2015).

PDT has attracted tremendous attention since its birth in the early 1900s, when Tappeiner conducted the first therapy to himself by topical eosin and sunlight. Later, Figger et al. and Lipson et al. discovered the photosensitizer hematoporphyrin and forwarded the PDT of the tumor to clinical applications, respectively (Figge et al., 1948). Compared with conventional approaches of cancer therapy such as surgical excision, chemotherapy, and radiotherapy, PDT shows great advantages such as higher spatial/temporal resolution and the anti-drug resistance.

As the photosensitizers may distributed over the whole body, a smart polymeric delivery system is beneficial for precise therapy. The delivery system is expected to protect the photosensitizers from systemic clearance, and carry the photosensitizer to the tumor site. In some cases, the delivery system releases the photosensitizers, either with a slow sustained release or a burst of release. Hence, the sustained release and biodegradable polymeric delivery systems are introduced. Smart polymeric delivery systems that can target the tumor and be activatable by the tumor microenvironment are highlighted.

### Sustained Release and Biodegradable Polymeric Delivery System

One of the most crucial functions of a polymeric delivery system is to enhance the water solubility of hydrophobic photosensitizers. For instance, polyethylene glycol (PEG) is one of the most frequently used polymers to increase the water solubility. PEG provides stealth to photosensitizers, making them invisible to microorganisms and cells. Hence the protection by PEG resisted protein adsorption and prolonged the circulatory half-life of photosensitizer (Keerthiga et al., 2020), which is beneficial to achieve long-term antitumor effects.

Polymers with high molecular weight may raise great concern about their toxicity. Hence, it is highly desired that the polymer can be degraded into small nontoxic fragments such as water and carbon dioxide, which is easy to be metabolized from the body (Kamaly et al., 2016). Concerning this aspect, biodegradable polymers such as polylactide (PLA), poly (glycolic acid) (PGA), poly (lactic-co-glycolic acid) (PLGA), and poly-β-benzyl-l-aspartate (PBLA) have been developed, which can be degraded slowly by the hydrolysis of the ester bond (Vert et al., 1992; Hoffman, 2008; Kim et al., 2009; Keerthiga et al., 2020) Hence the encapsulated photosensitizers can be sustained released.

### Tumor-Target Polymeric Delivery System

#### Passive Targeting

The passive targeting of those polymeric nanoparticles is referred to the enhanced permeation and retention (EPR) effect proposed by Hiroshi Maeda in Kumamoto, Japan, with the theory based on the high permeability of blood vessels at tumor sites, due to the leakage of the tumor vasculature as a consequence of growing fast (Hoffman, 2008). There are plenty of systems that enhance the accumulation efficacy to tumor *via* EPR effect (Regehly et al., 2007; Lee et al., 2009). However, this targeting strategy may release a considerable quantity of drugs before substantial uptake by tumor cells. Moreover, recently the EPR effect is becoming a controversial topic as researchers found that human bodies do not have obvious leakage of tumor vasculature and EPR effect. Hence, the general applicability of this EPR effect is challenged and still an open question.

#### Active Targeting

Active targeting includes the use of targeting moieties such as ligands, antibodies, and aptamers that can recognize specific receptors on tumor vasculature, tumor cells, and even tumor subcellular organelles for enhanced delivery of photosensitizers (Li and Yan, 2018; Zhang et al., 2020; Zheng et al., 2020)

The tumor vasculature targeting is an efficient way to combat cancer as a result of cutting off the supply of oxygen and nutrients. In addition, by interrupting the tumor vessel integrity, an increased accumulation of the materials to the tumor tissue is expected to improve the PDT efficacy. Vascular endothelial growth factor (VEGF) and its receptor are hallmarks of tumor cells, which can be utilized as main antiangiogenic targets (Tirand et al., 2006; Prasad et al., 2014). Vascular cell adhesion molecule-1 (VCAM-1) is involved in tumor cell adhesion and metastasis (Yin et al., 2017), and matrix metalloproteinases (MMPs) are responsible for the angiogenesis and metastasis (Danhier et al., 2010; Liu T. W. et al., 2016), RGD ligands are another frequently used target and the recognition is *via* α_v_β_3_ integrin (Yuan et al., 2014). All of them are crucial tumor vasculature targets.

Target tumor cells are a straightforward targeting strategy that accelerate the phagocytosis/endocytosis of nanocarriers (Hanahan and Weinberg, 2011; Liu L. et al., 2016; Liu et al., 2017). The representative receptors are CD44 receptor (Jiang et al., 2017), folate (FA) receptor (Li et al., 2018d), transferrin receptor (Kaspler et al., 2016), epidermal growth factor (EGF) receptor (del Carmen et al., 2005), etc.

Subcellular targeting, such as mitochondria, lysosome, nucleus, and endoplasmic reticulum, is another type of targeting. Among these, mitochondria targeting is of great importance as mitochondria is the energy factory of tumor cells (Chakrabortty et al., 2017). The triphenylphosphonium (TPP) cation is a typical mitochondria targeting moiety. Due to the limited diffusion distance of singlet oxygen, the subcellular targeting of different organelles may lead to varying degrees of destruction of tumor cells.

**FIGURE 2:**
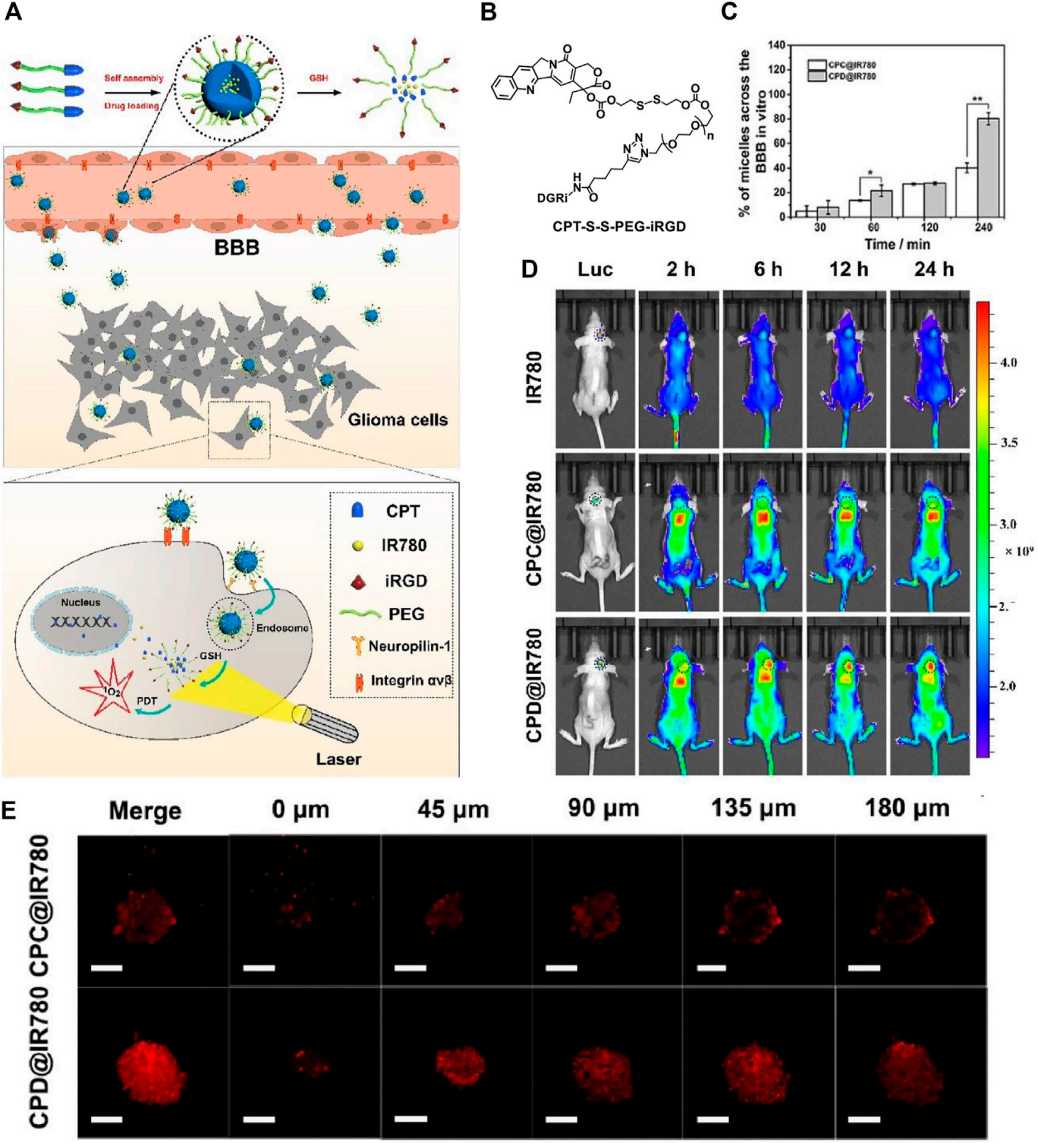
**(A)** Preparation of self-assembled micelles, BBB penetrating, and glioma cell targeting by the iRGD peptide and GSH-induced disassembly for the combination of chemotherapy and photodynamic therapy; **(B)** molecular structure of CPT-S-S-PEG-iRGD; **(C)** the quantitative analysis of absorption spectra in the lower chamber after CPC@IR780 micelles and CPD@IR780 micelles were cultured in the upper chamber for different times; **(D)** whole body fluorescence imaging of U87 orthotopic glioma mice at different time intervals. Dashed line indicates the glioma site; **(E)** intratumoral penetration characterization using U87 tumor spheroids treated with micelles in 45 μm interval between consecutive slides. Reprinted with permission from Lu L. et al. (2020).

With a large amount of targeting candidates mentioned above, herein we only choose one example for the demonstration of the importance of targeting function in PDT. Target function is especially crucial to cross the blood-brain-barrier (BBB) in the treatment of brain tumor. The treatment of brain disease is not a trivial task as the delivery of the drugs to the brain area is very difficult due to the BBB. An iRGD-conjugated prodrug micelle with BBB penetrability was used to enhance the anti-glioma therapy (Lu L. et al., 2020) As shown in Figure 2, the structure of the polymer CPT-S-S-PEG-iRGD consists of a prodrug camptothecin (CPT) for chemotherapy, a disulfide linker responsive to glutathione (GSH), and the internalizing RGD peptide (iRGD) as targeting moiety. The ability to cross the BBB and target glioma cells is expected to be enhanced *via* αvβ integrin and neuropilin-1 mediated ligand transportation. The polymer is self-assembled into micelle with a diameter of approximately 100 nm. A photosensitizer IR780 is loaded into the micelle for PDT.

Hence, the micelle is functionalized as the CPT and photosensitizer IR780 carrier, which is responsive to the high concentration of GSH in glioma tumor and triggers the release of the therapeutics CPT and IR780. The iRGD peptide bonded at the surface of the micelles is responsible for the cross of the BBB and glioma tumor targeting. The penetration ability of the micelles is examined *in vitro* BBB model, which is established by co-culture of bEnd.3 cells and U87 cells in the upper and lower chamber, respectively. The bEnd.3 simulates the BBB. By examination of the uptake efficiency of the micelles by U87 cells in the lower chamber, the penetration ability of the materials is found to be significantly improved (Figure 2C). The results show that 89.92 ± 1% of iRGD modified CPD@IR780 micelles cross the simulated BBB into the lower chamber, while for the unmodified CPC@IR780 micelles the value is only 41.78 ± 0.22%.

The authors further constructed U87 tumor spheroids *in vitro* to examine the penetration ability (Figure 2E). For the CPD@IR780 micelles, the fluorescence is stronger and uniformly distributed in the glioma spheroids. While for the CPC@IR780 micelles, the fluorescence is mainly distributed in the periphery of the glioma spheroids. These results demonstrated the enhanced penetration ability to the glioma spheroids of **CPD@IR780** micelles due to iRGD modification. The biodistribution of micelles *in vivo* was studied (Figure 2D). The iRGD modified CPD@IR780 micelles show enhanced accumulation in the brain, as compared to the unmodified CPC@IR780 micelles and IR780. Confocal images of brain sections of U87 orthotopic glioma mice were studied. The iRGD modified CPD@IR780 micelles show preferably distribution in glioma tumor cells instead of healthy cells. All those results confirmed the target ability of CPD@IR780 micelles to glioma cells. The combination of activatable chemotherapy and PDT with target function to enhance penetration to BBB successfully extended the survival time of mice bearing glioma tumor.

### Tumor-Activatable Polymeric Delivery System

Abnormal expression of proteins usually has a close relationship with diseases. Enzymes are especially crucial biomarkers, and a variety of enzymes are overexpressed by tumor cells or tumor associated macrophages. For instance, hyaluronidase, matrixmetalloproteinases, and cathepsin B are overexpressed in tumor cells. The enzyme-activated polymeric delivery systems have been developed, which have high specificity as compared with other activation strategies such as pH and GSH activation (Bae and Na, 2010; Park W. et al., 2011; Li H. et al., 2017; Huo et al., 2019).

**FIGURE 3:**
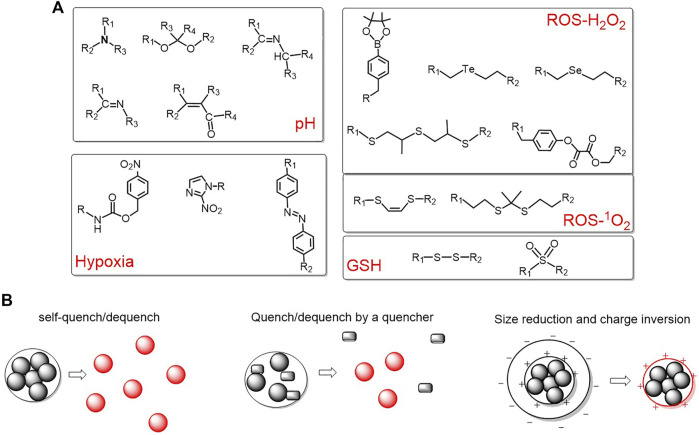
**(A)** Representative pH/ROS/GSH/hypoxia-responsive linkers for activatable polymeric delivery system; **(B)** three commonly used activation strategies.

The increased glycolysis and proton-pump activities on plasma membranes of cancer cells produce a large amount of lactic acid, resulting in a slightly acidic extracellular microenvironment of tumor. The pH value of tumor sites is in the range of 6.0–7.0, which is lower than that of normal tissues (pH = 7.4) (Pan et al., 2013). Taking advantage of the difference in pH, an acid activatable system can be designed (Du et al., 2010; Du et al., 2011; Yuan et al., 2012; Tseng et al., 2015; Li F. et al., 2018; Dong et al., 2018; Lin et al., 2019; Lu Y. et al., 2020) mainly based on the protonation of the amine group or acid cleavage of acidic-labile linkers such as hydrazone linkers (Braslawsky et al., 1990), Schiff base linkers (-RC = N-) (Ke et al., 2014), *cis*-acotinyl linkers (Zhu et al., 2010), acetal linkers (Gu et al., 2013), etc. (Figure 3A).

Reactive oxygen species (ROS) and GSH are of great importance for maintaining the redox stability. Tumor cells have higher levels of ROS due to the oncogenic stimulation, mitochondrial malfunction, and increased metabolic activity(Li X. et al., 2017). For instance, the concentration of hydrogen peroxide in tumor cells is reported to be approximately 10 μM, which is higher than normal cells. Boronic acid esters are typical hydrogen peroxide-responsive moieties (Saravanakumar et al., 2017; Zeng et al., 2020). In addition, GSH is abundant in a tumor intracellular environment (2–10 mM) (Li and Yan, 2018). Disulfides and sulfonyl group are frequently utilized to construct GSH-responsive polymeric delivery systems (Kim et al., 2014; Li et al., 2014; Liu X. et al., 2016). The representative ROS and GSH responsive organic moieties are summarized in Figure 3A.

Due to the consumption of oxygen within about 100 µm of the inadequate tumor vasculatures by quickly proliferating cancer cells, the oxygen concentration of tumors is only about 4%, which is very distinct from normal tissues. Based on this, hypoxia-responsive polymeric systems have been developed and three representative hypoxia-responsive moieties have been summarized in Figure 3A.

Based on the different microenvironments of tumor and normal tissues, a variety of activatable polymeric delivery systems are emerging (Park et al., 2016; Sun et al., 2017; Wei et al., 2018; Li et al., 2019c; Yan et al., 2020; Zeng et al., 2020; Yang et al., 2021). Despite the different structures and constituents of thousands of reports, they mainly achieve the activation *via* the following three strategies: (1) self-quenching and dequenching of photosensitizers due to aggregation and disintegration; (2) utilize another quencher to quench the triplet state of photosensitizer and dequenching upon cleavage of sensitive bonds; and (3) the change of size and surface charge to induce an enhanced internalization by tumor cells. These three strategies are summarized in Figure 3B.

Herein we introduce some typical polymeric delivery systems for PDT, as an illustration of the above-mentioned activations and strategies.

#### Enzyme

**FIGURE 4:**
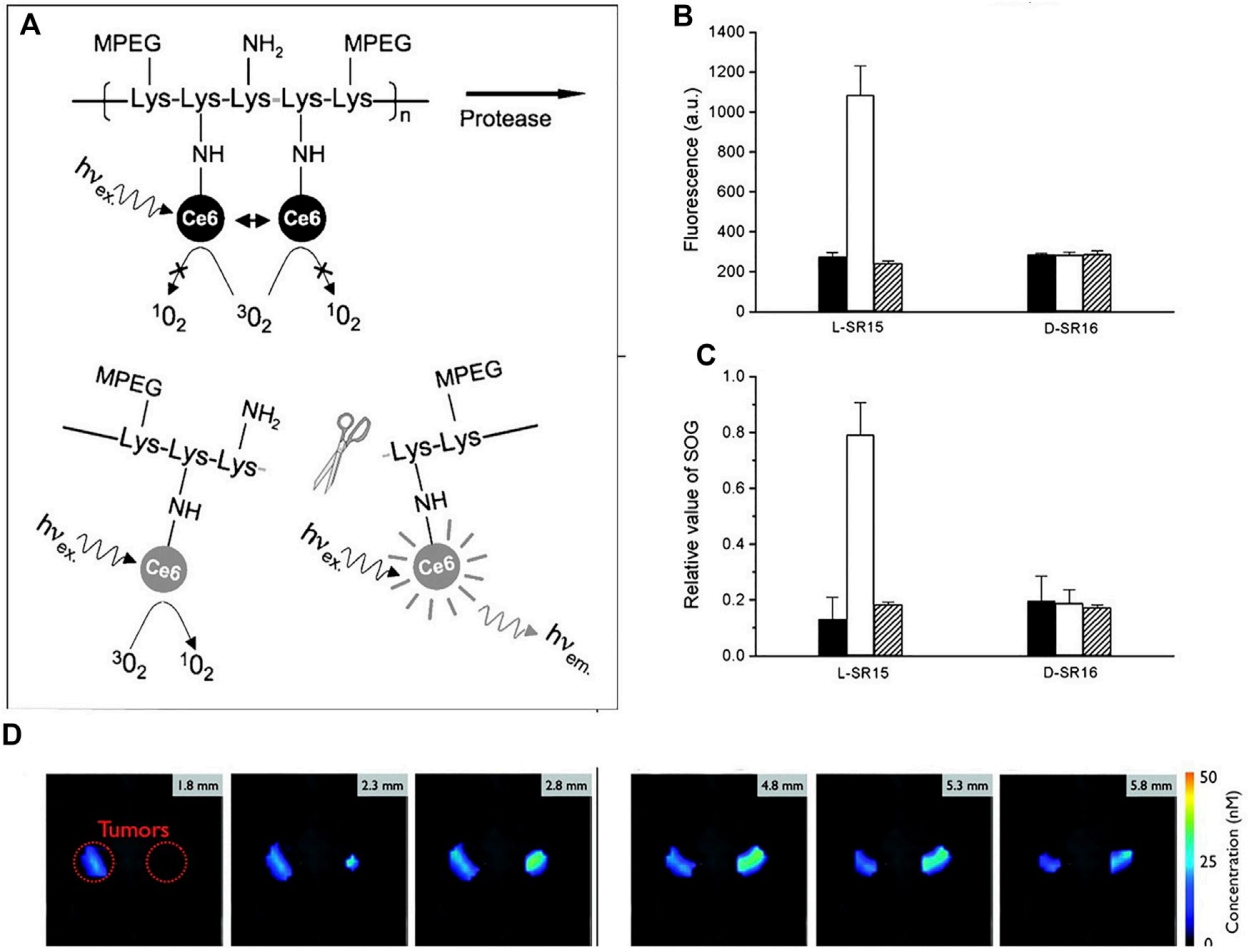
**(A)** Schematic diagram of PM-PDT strategy; **(B)** activation of fluorescence intensity and **(C)** singlet oxygen generation of L-SR15 and D-SR16 with phosphate buffer (black columns), cathepsin B (white columns), and CA-074 inhibitor-pretreated cathepsin B (striped columns); **(D)** six consecutive slices from a three-dimensional fluorescence-mediated tomographic scan (Choi et al., 2006).

Choi et al. reported a protease biodegradable poly-L-lysine, grafted with PEG and photosensitizer Ce6 (L-SR15). As shown in Figure 4, the quenched fluorescence and singlet oxygen production ability is recovered due to the degradation of the poly-L-lysine by tumor-associated protease cathepsin B. A poly-D-lysine backbone (D-SR16) that is uncleavable by proteases was synthesized for comparison. *In vitro* results are shown in Figures 4B,C, both the fluorescence/singlet oxygen generation ability of L-SR15 and D-SR16 are significantly decreased as compared with the free Ce6. The fluorescence intensity/singlet oxygen generation ability of L-SR15 is recovered by the addition of cathepsin B, while there is no change for D-SR16. The fluorescence/singlet oxygen generation ability enhancement can be inhibited by the addition of cathepsin B inhibitor CA-074. *In vivo* results show that the tumor can be imaged by the L-SR15 (Figure 4D), due to the selectively activation by the cathepsin B in the tumor site. The tumor volume is significantly depressed for the L-SR15 group as compared with D-SR16 group or L-SR15 + inhibitor CA-074 group. These results demonstrated that the tumor-specific protease cleaved the poly-L-lysine backbone, restoring the self-quenched fluorescence/singlet oxygen production ability of Ce6 and achieving selective imaging and PDT of the tumor.

A similar strategy was reported by Kun Na et al., who is also one of the pioneer researchers in this area. For instance, photosensitizer Pheophorbide (Pba) was dangled onto hyaluronic acid (HA), which leads to self-quenching of the photosensitizer. The polymer can be easily internalized by HA receptor-mediated endocytosis and then degraded by intracellular microenvironment enzymes, releasing free Pba with recovered PDT efficacy (Li et al., 2010). However, those reports by Choi et al. and Kun Na et al. are based on the self-quenching of photosensitizers. Both the quenching and activation efficiency are highly dependent on the substitution degree of the photosensitizers to the polymer backbone. A higher degree of substitution will lead to higher quenching efficiency but maybe lower activation efficiency because it decreases the recognition site of the biomarker.

**FIGURE 5:**
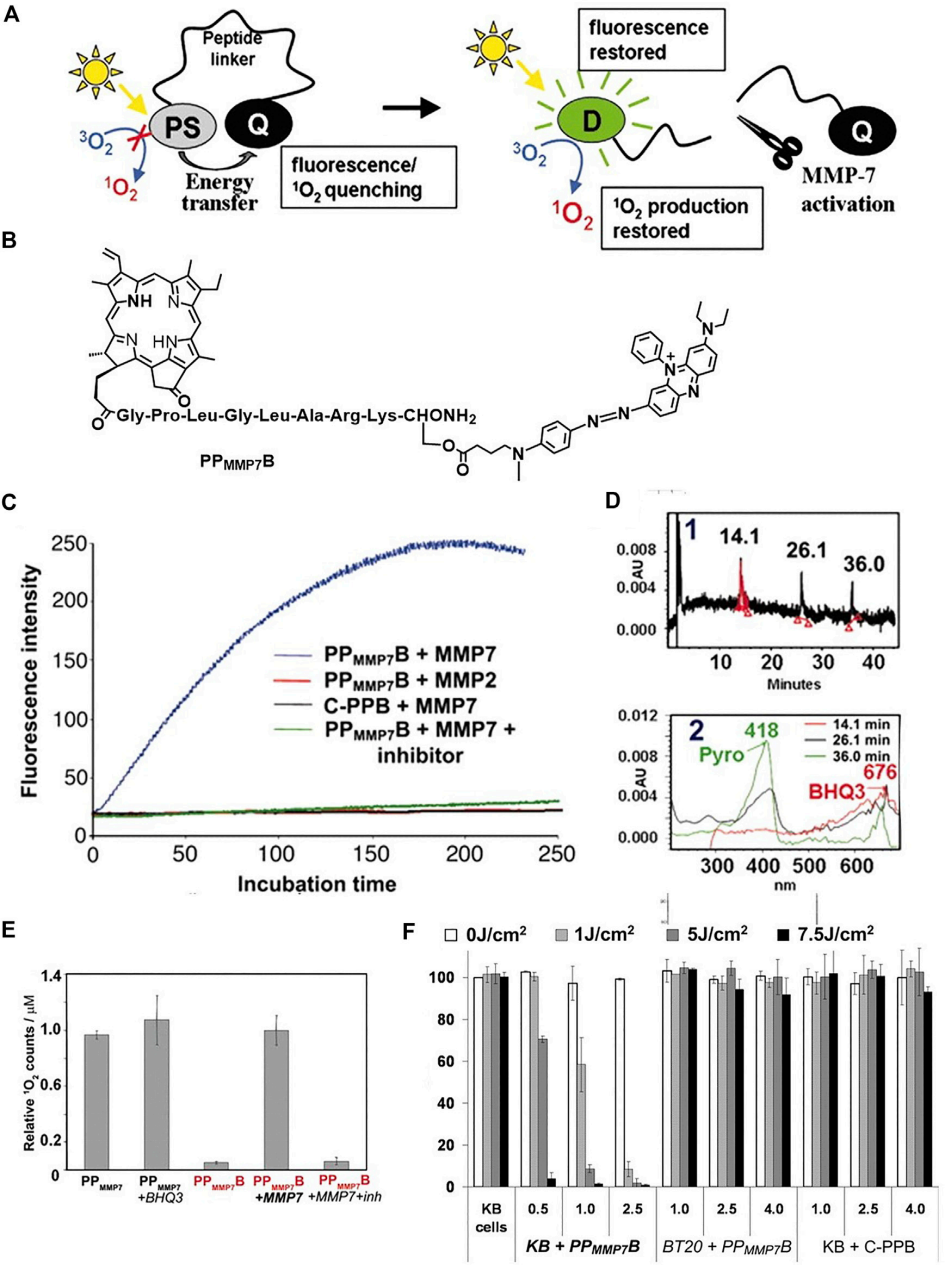
**(A)** The concept of photodynamic molecular beacons. **(B)** Molecular structure of PP_MMP7_B. **(C)** Fluorescence kinetics of different groups. **(D)** HPLC spectrum of **PP**_**MMP7**_ **B** + MMP7 incubated at 37°C for 2 h and corresponding UV-vis spectra. **(E)** The relative ^1^O_2_ counts of different groups. **(F)** Photodynamic cytotoxicity determined by MTT assay as a function of PS and light doses, compared with untreated cells. Copyright (2007) National Academy of Sciences (Zheng et al., 2007).

Zheng et al. reported a molecular beacon system based on intramolecular quenching (Zheng et al., 2007). As shown in Figure 5A, triplet photosensitizer (pyropheophorbide, Pyro) is connected with a quencher (BHQ3) by a peptide linker, which is activatable by tumor-associated protease MM7. Both the fluorescence and the singlet oxygen production ability of Pyro can be quenched by BHQ3, while it restored upon cleavage of the peptide linker by the MM7. This is confirmed with fluorescence emission and the direct detection of singlet oxygen (Figure 5C and Figure 5E). The molecular beacon is specifically cleavable by MM7 peptide (PP_MMP7_B + MMP7 in Figure 5C), while uncleavable by MM2 peptide (PP_MMP7_B + MMP2 in Figure 5C). Moreover, the addition of MM7 inhibitor (PP_MMP7_B + MMP7+inhibitor in Figure 5C) cannot restore the fluorescence and singlet oxygen production ability. All these results demonstrate the selectivity of the peptide linker to MM7. The cleavage of the peptide linker was further evidenced by high performance liquid chromatography (HPLC) in Figure 5D. The PP_MMP7_B has a retention time of approximately 26.1 min. The cleavage of PP_MMP7_B by MM7 resulted in two fragments peaked at 14.1 and 36.0 min, corresponding to the BHQ3 moiety and Pyro moiety, respectively, which is confirmed by the UV-vis absorption spectroscopy and MALDI-TOF mass spectroscopy. The PP_MMP7_B shows significant photodynamic cytotoxicity (Figure 5F) in KB cells (MMP7 overexpressed cell line) compared with the BT20 cells (MMP7 less expressed cell line). The KB tumors are found to be greatly depressed by the enzyme cleavable molecular beacon PP_MMP7_B.

#### pH

**FIGURE 6:**
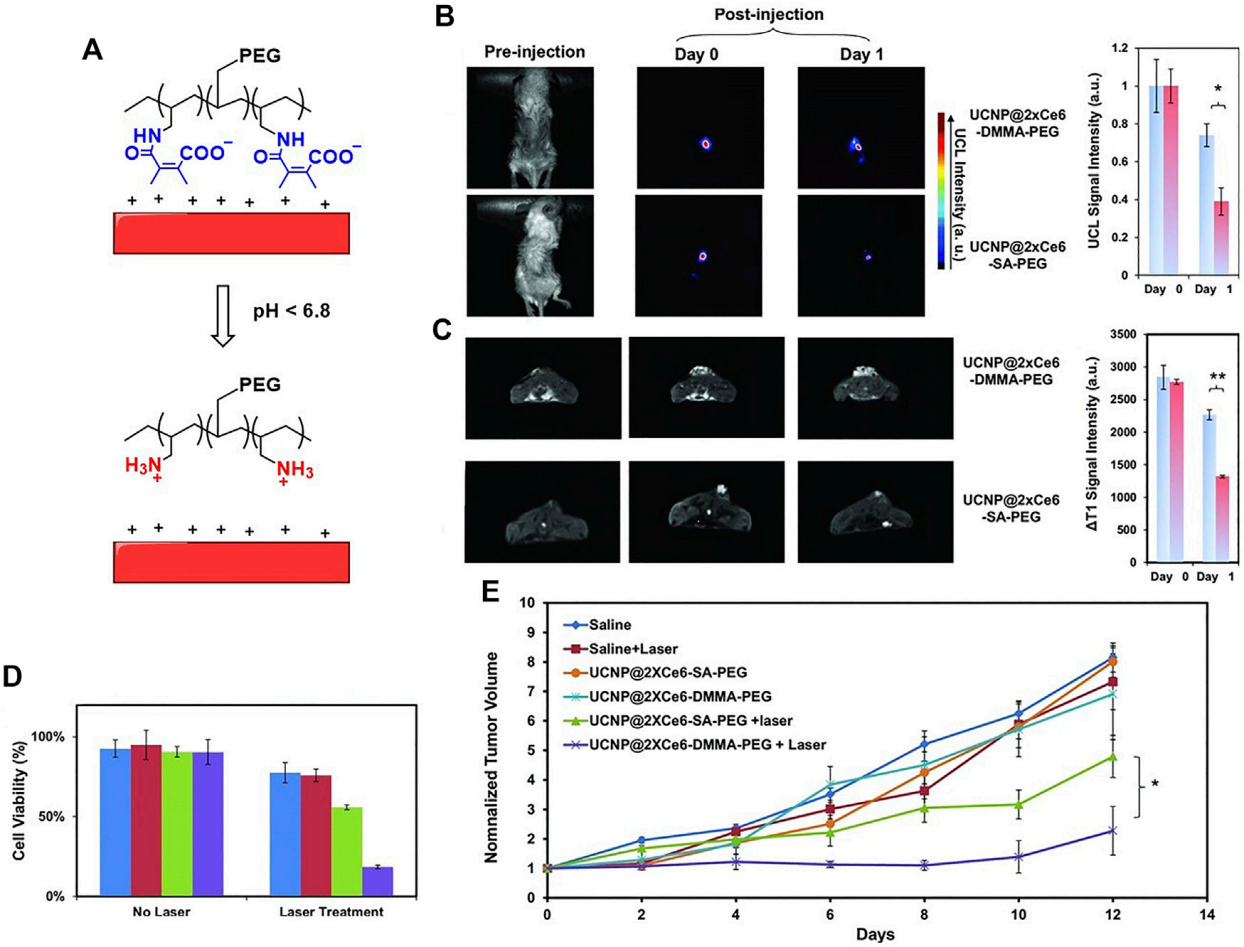
**(A)** Schematic illustration of pH-responsive smart theranostic UCNPs, showing the detachment of PAH-DMMA-PEG from the positively charged nanoparticle surface under pH 6.8; **(B)** *in vivo* UCL images and **(C)** T_1_ MR images of mice after intratumoral injection with UCNP@2xCe6-DMMA-PEG or UCNP@2xCe6-SA-PEG. The images were taken at different times post-injection; **(D)** cell viability data of HeLa cells after various treatments indicated with and without the 980 nm laser irradiation as evaluated by the standard MTT assay; **(E)** tumor growth curves of different groups of mice after various treatments. Reprinted with permission from Wang et al. (2013).

Liu et al. constructed an imaging-guided pH sensitive PDT platform by using charge reversible up-conversion nanoparticles (Wang et al., 2013). As shown in Figure 6A, the pH-sensitive polymer PAH-DMMA-PEG (negatively charged) was grafted onto the positive surface of the Mn^2+^-doped, Ce6 layered up-conversion nanoparticles (UCNPs) *via* electrostatic interactions (UCNP@2XCe6-DMMA-PEG). At pH = 6.8, the nanoparticle zeta potential increasing from approximately −18 mV to +10 mV, confirming the acid-responsive detaching of the PEG coating from the UCNPs. UCNP@2XCe6-SA-PEG that is not responsive to acid is used as a reference. As expected, the zeta potential remained the same in pH = 7.4 and pH = 6.8. The ability of the internalization by cancer cells at different pH was studied by confocal laser scanning microscopy imaging. The UCNP@2XCe6-DMMA-PEG at pH = 6.8 shows increased internalization ability than at pH = 7.4, hence enhanced phototoxicity to HeLa cells was observed (Figure 6D). Owing to the doping of the Mn^2+^ to the UCNPs, the materials can be used for dual-modal imaging, i.e., up-conversion luminescence imaging and magnetic resonance imaging (Figure 6B and Figure 6C). Both materials show good accumulation in tumor sites. UCNP@2XCe6-DMMA-PEG has a longer retention in tumor than the reference UCNP@2XCe6-SA-PEG. This is due to the acid-responsive detaching of the PEG coating resulting in a strong positive surface of UCNPs, which enhanced the stickiness of the material to the negatively charged cancer cell membranes and tumor tissues. The longer retention time of UCNP@2XCe6-DMMA-PEG contributes to the better PDT efficacy *in vivo* than UCNP@2XCe6-SA-PEG (Figure 6E).

#### ROS

**FIGURE 7:**
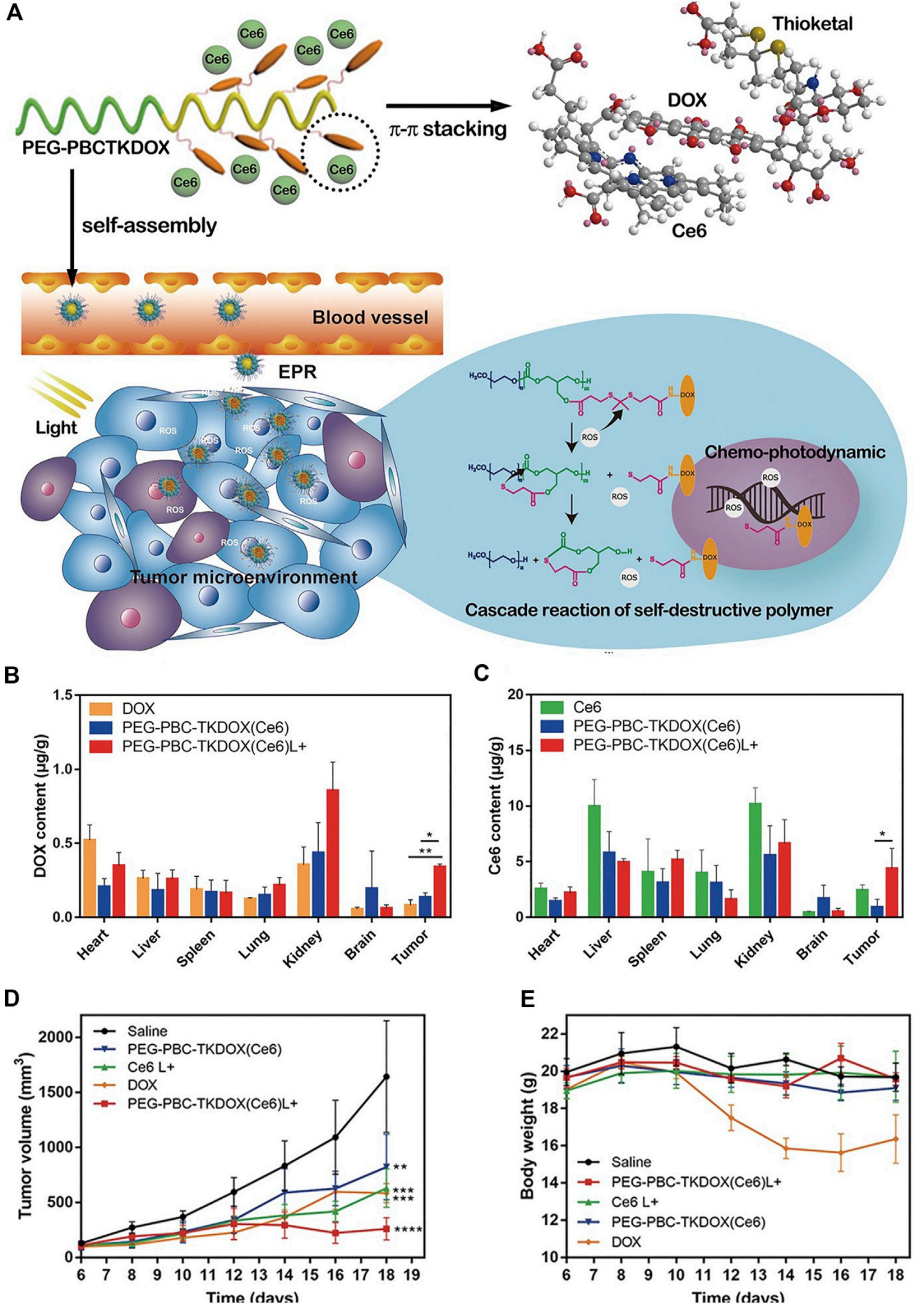
**(A)** Schematic drawing of the cascade reaction of self-destructive polymeric nanomicelles; **(B)** biodistribution of DOX solution and PEG-PBC-TKDOX (Ce6) micelles at 12 h with or without light irradiation; **(C)** biodistribution of Ce6 solution and PEG-PBC-TKDOX (Ce6) micelles at 12 h with or without light irradiation; **(D)** growth curves of subcutaneously inoculated KB tumors. Intravenous injections were given to mice at day 7, day 9, and day 11. The 660 nm laser irradiation followed at 12 h after each injection; **(E)** body weight changes (*n* = 5, **, *p* < 0.01, ***, *p* < 0.001, ****, *p* < 0.0001). Reprinted with permission from Wang et al. (2019).

An ROS responsive self-degrading polymer was constructed for enhanced chemotherapy and PDT (Wang et al., 2019). As shown in Figure 7A, the therapeutic prodrug DOX was conjugated to the polymer with a pendant thioketal bond, which is responsive to ROS such as singlet oxygen or hydrogen peroxide. The triplet photosensitizer Ce6 with a planar aromatic structure interacted with the DOX *via* π-π stacking, constituting a self-assembled nanoparticle PEG-PBC-TKDOX(Ce6). In the tumor microenvironment or upon light irradiation, the abundant hydrogen peroxide in the tumor microenvironment or the singlet oxygen produced from irradiated Ce6 leads to the cleavage of the pendant thioketal bond, followed by the cascade reaction of self-destructive polymer. Accordingly, the Ce6 and DOX are actively released (Figure 7A). The degradation of the polymer was confirmed by ^1^H-NMR characterization and the decrease of the molecular weight in the presence of hydrogen peroxide. The release of DOX was confirmed by confocal laser scanning microscopy imaging. Free DOX has fluorescence and accumulates in nuclear. As compared with those without light irradiation, KB cells incubated with PEG-PBC-TKDOX(Ce6) upon light irradiation show enhanced accumulation in nuclear, indicating the release of DOX from polymer backbone. Biodistribution of DOX and Ce6 was studied (Figures 7B,C). The light irradiation enhanced the accumulation of the PEG-PBC-TKDOX(Ce6) in tumor sites. This may be attributed to the ROS induced destruction of the polymer, as smaller molecular size is known to have better tumor accumulation ability (Wang et al., 2019). The PEG-PBC-TKDOX(Ce6) upon light irradiation shows outstanding therapeutic efficacy (Figure 7D) and decreased free DOX-related side effects such as decrease of body weight (Figure 7E).

#### GSH

**FIGURE 8:**
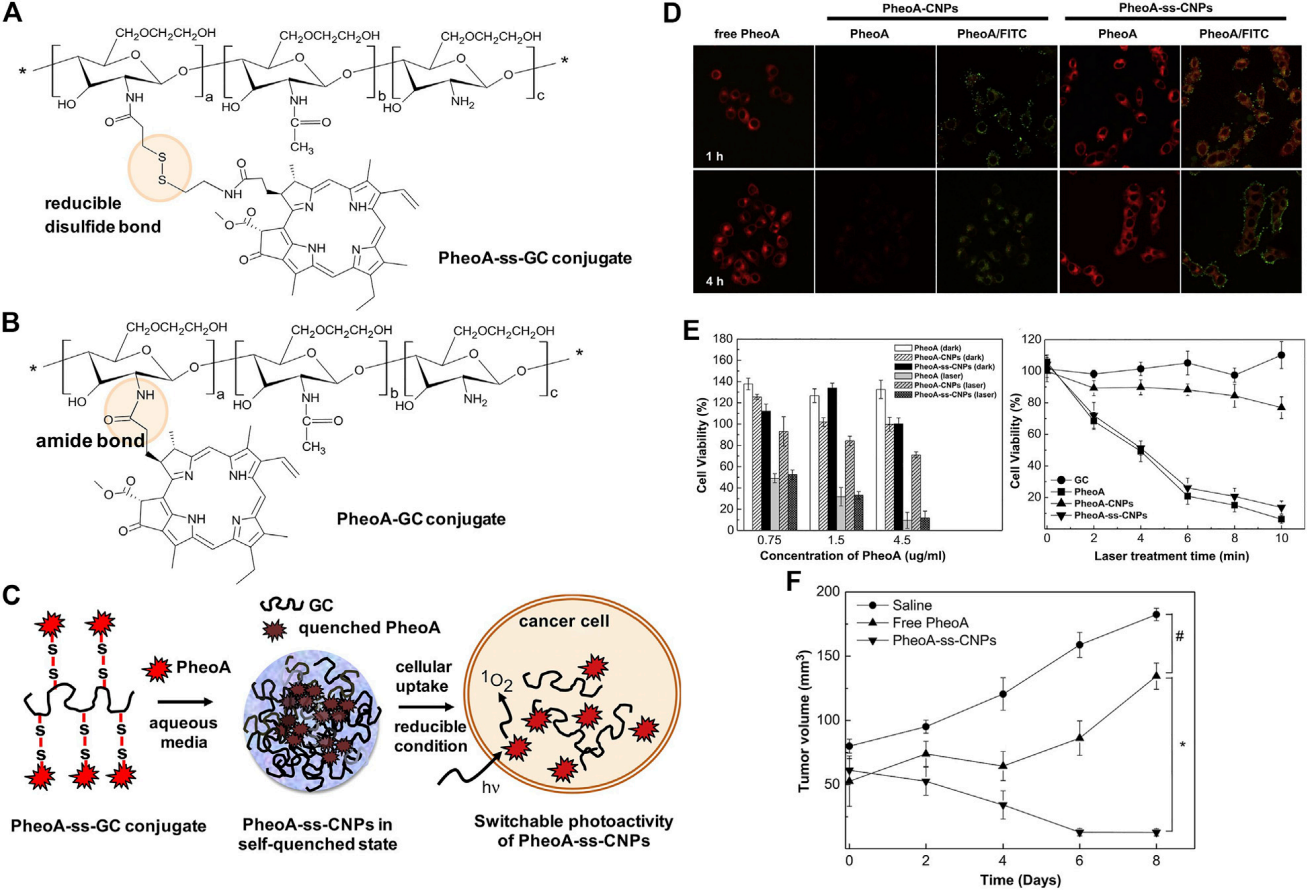
Chemical structures of **(A)** PheoA-ss-GC and **(B)** PheoAeGC conjugates and **(C)** illustration of bioreducible PheoA-ss-CNPs for switchable photoactivity of PheoA; **(D)** CLMS images of KB cells treated with free PheoA, PheoAeCNPs, and PheoA-ss-CNPs; **(E)** phototoxicity and dark toxicity of free PheoA, PheoAeCNPs, and PheoA-ss-CNPs; **(F)** tumor growth of HT-29 tumor-bearing mice after various treatments. Reprinted with permission from Oh et al. (2013).

Natural polysaccharides have been popularly used for the development of self-quenchable nanoparticles due to their good water solubility, biodegradability, biocompatibility, and tumor targeting ability (Bae et al., 2010; Bae and Na, 2010; Li et al., 2011; Yoon et al., 2012) Kang Moo Huh et al. constructed a GSH responsive pheophorbide a-glycol chitosan system for PDT. As shown in Figure 8C, the photosensitizer pheophorbide a (PheoA) was dangled onto the glycol chitosan *via* a reducible disulfide bond (PheoA-ss-GC in Figure 8A), which is sensitive to the GSH that is abundant in the tumor microenvironment. The conjugate tends to aggregate into nanoparticles and the fluorescence/singlet oxygen production ability of PheoA is diminished due to self-quenching. The cleavage of the disulfide bond by GSH recovered both the fluorescence and singlet oxygen production ability, achieving the on-site release of the photosensitizer and selectively killing tumor cells. A reference compound (PheoA-GC in Figure 8B) that has no disulfide bond linker was also synthesized for comparison. The recovery of fluorescence and singlet oxygen production ability of PheoA-ss-GC were evidenced both in cuvette and *in vitro*. As shown in Figure 8D, the fluorescence of PheoA is obvious in the KB cells treated with PheoA-ss-GC, while it was completely quenched in the cells treated with PheoA-GC. Moreover, the PheoA-ss-GC shows significantly higher phototoxicity to KB cells than PheoA-GC (Figure 8E) as the self-quenching effect of the photosensitizer is greatly alleviated upon the activation of high concentration of GSH in tumor cells. Consequently, outstanding PDT efficacy of PheoA-ss-GC is achieved (Figure 8F).

#### Hypoxia

Tumor tissues are known to be hypoxic. Taking advantage of this characteristic, Zhao et al. designed multifunctional micelles that are dually responsive to hypoxia and singlet oxygen (Li et al., 2018c). The molecular structure is consisted of azobenzene and imidazole. The hypoxic atmosphere of the tumor tissues induces the collapse of azobenzene and consequently provoked PEG shedding (dePEGylation); the singlet oxygen produced by Ce6 upon light irradiation leads to an oxidation of hydrophobic imidazole to hydrophilic urea, followed by a rapid release of Ce6. Enhanced internalization of micelles by LLC cells and improved PDT efficacy is observed.

#### Temperature

Besides the internal triggers such as pH, enzyme, GSH, and hypoxia, external triggers such as temperature can also activate the PDT. Poly (N-isopropylacrylamide) (PNIPAM) is a frequently used thermo-sensitive polymer (Liu et al., 2009; Lv et al., 2015). It can go through the phase transition from hydrophilic to hydrophobic state when the temperature is above the lower critical solution temperature (LCST) (Stöber et al., 1968; Pelton and Chibante, 1986; Pelton, 2000). The LCST of PNIPAM in water is approximately 32–33°C. Molecular structure modification to the PNIPAM (co-polymerization with other monomers) or adjusting the pH value can affect this LCST value (Hoare and Pelton, 2004; Rijcken et al., 2007; Dai et al., 2012; Zhang et al., 2013; Jiang et al., 2014). Hence it is possible to adjust this LCST to suitable values such as body temperature (ca. 37°C). In addition, tumor tissues are reported to have a higher temperature than normal tissues due to inflammation and cancer cell immortalization (Wang et al., 2013; Lv et al., 2015)

**FIGURE 9:**
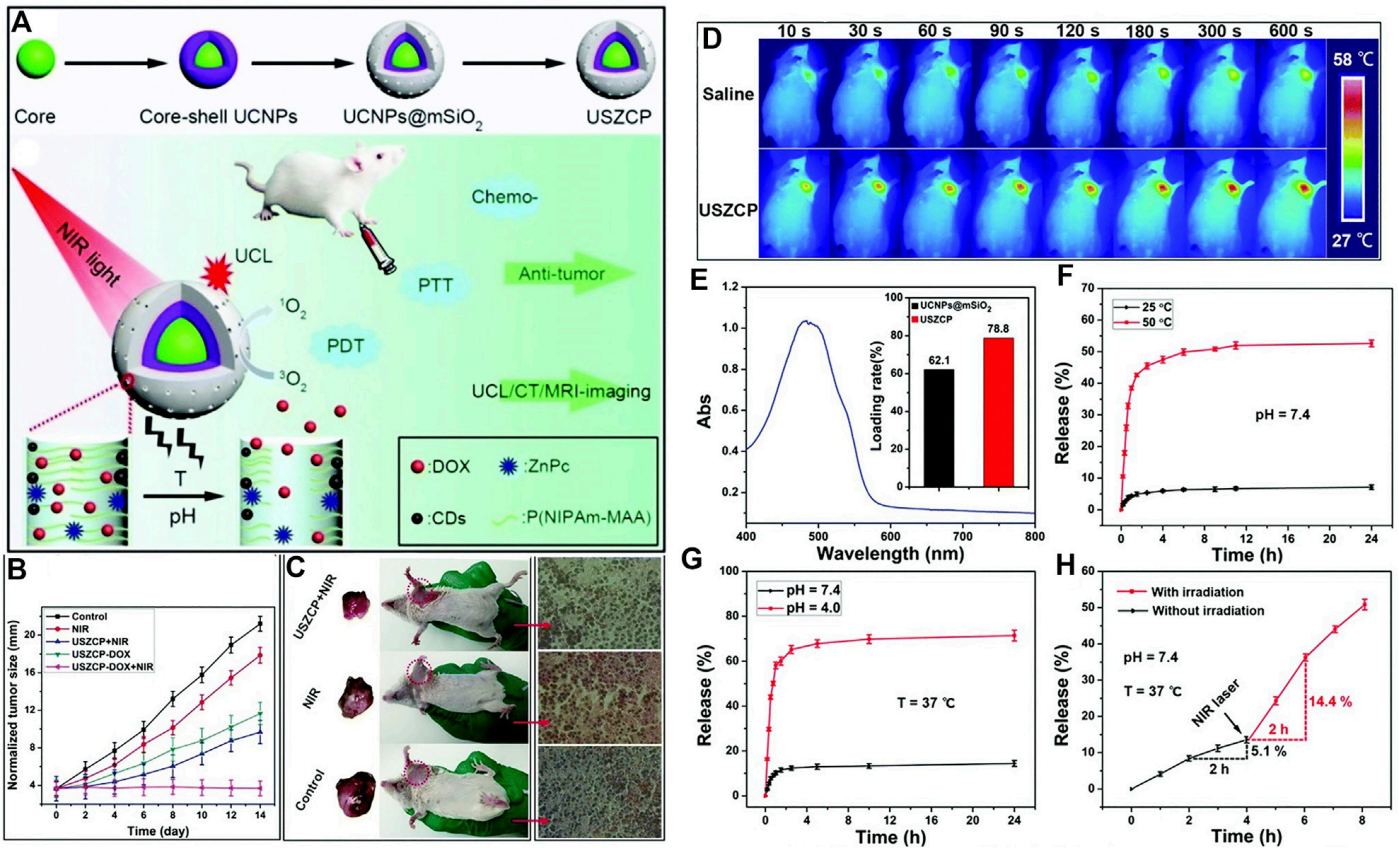
**(A)** Schematic illustration of pH-temperature sensitive polymer for cancer therapy. **(B)** Changes in the tumor size of the H22 tumor obtained from mice after different treatments; **(C)** Picture of tumor and H&E staining; **(D)** *in vivo* infrared thermal images of a tumor-bearing mouse after injection of USZCP under NIR irradiation with different times; **(E)** UV-vis absorption spectrum of DOX (inset shows the DOX loading content of UCNPs@mSiO2 and USZCP); the release efficiency of USZCP–DOX at different **(F)** temperatures and **(G)** pH values; **(H)** the release efficiency of USZCP–DOX triggered by a NIR laser. Reprinted with permission from Xu et al. (2016).

A nanoplatform based on the pH-temperature sensitive polymer was constructed for cancer therapy (Xu et al., 2016). As shown in Figure 9, the up-conversion nanoparticles (UCNP) are decorated with carbon dots and chemotherapeutic reagent DOX and PDT reagent zinc(II)-phthalocyanine (ZnPc). The up-converted fluorescence excited the ZnPc to its triplet state and produce singlet oxygen, and the decorated carbon dots generate photothermal effect upon near infrared laser irradiation. This thermal effect at tumor acidic tissues induces the shrinkage of P(NIPAm-MAA) polymer, leading to the controlled drug release. As shown in Figure 9F, the nanoplatform shows significant drug release at 50°C (pH = 7.4). Moreover, the drug can be efficiently released at acidic conditions (pH = 4.0, Figure 9G) at 37°C. These results show that the drug release can be controlled by pH and temperature. The thermal effect of the nanoplatform upon NIR laser irradiation was shown in Figure 9D. The NIR laser irradiation to the material accumulated in tumor site increased the local temperature, hence the drug release upon NIR laser irradiation can be expected (Figure 9H). The combination of the PDT and chemotherapy shows an outstanding therapeutic efficacy (Figure 9B and Figure 9C).

## Smart Polymeric Delivery System for Antimicrobial Photodynamic Therapy

Antibiotics have been the most effective strategies to fight against bacterial infections. However, the abuse of antibiotics has led to severe antimicrobial resistance (Bush et al., 2011). There are approximately 700,000 deaths per year due to antimicrobial resistance all over the world (Klausen et al., 2020). Developing other anti-bacterial strategies such as PDT that are alternatives to antibiotics is of great urgency (Demidova and Hamblin, 2004; Hamblin and Hasan, 2004; Pagonis et al., 2010; Huang et al., 2012).

**FIGURE 10:**
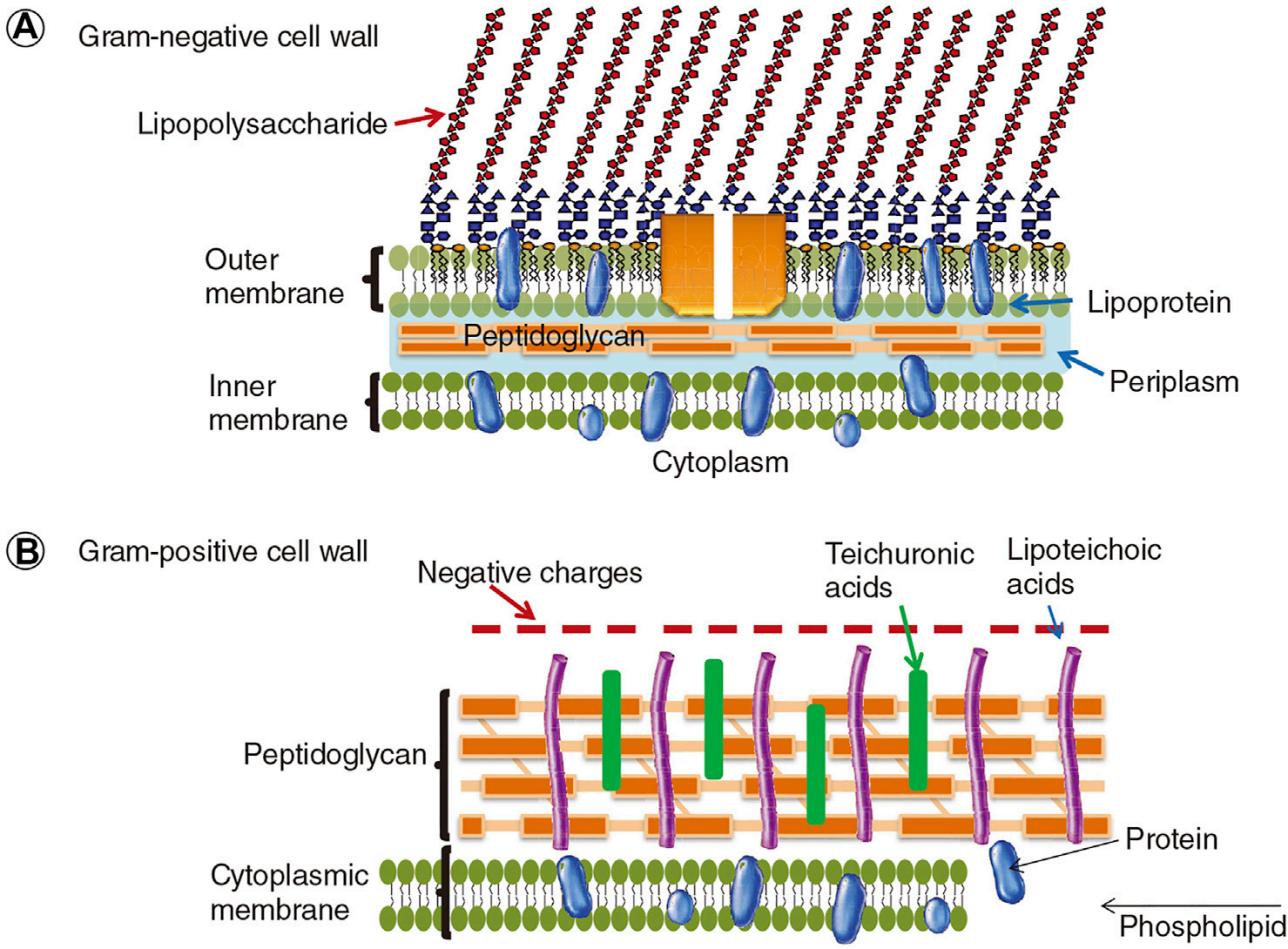
The cell walls of Gram-negative and Gram-positive bacteria. Reprinted with permission from Yin et al. (2015).

The delivery of the photosensitizer to the bacteria (especially Gram-negative bacteria) is not a trial task due to its structural features. As shown in Figure 10, the cell wall of Gram-positive bacteria consists of thick, porous layers of peptidoglycan embedded with proteins and lipoteichoic acid. The porous layers are relatively easy to go through for photosensitizers. Moreover, the lipoteichoic acids on the outside are negatively charged, which tends to bind with cationic agents (Minnock et al., 2000; Lambert, 2002). As a comparison, the cell wall of Gram-negative bacteria is thicker, being composed of a peptidoglycan layer, the inner cytoplasmatic membrane, and the outer membrane. This additional outer membrane of Gram-negative bacteria is composed of phospholipids and lipopolysaccharides, which is an effective barrier that limits the penetration of photosensitizers (Minnock et al., 2000). Hence, a smart polymeric delivery system that can target the bacteria and selectively kill the bacteria is necessary for optimal antimicrobial PDT.

### Bacterial-Target Polymeric Delivery System

#### Structure-Inherent Targeting

As it has been mentioned that the structures of the tumor cells and the bacteria are different, photosensitizers that are commonly used in PDT of tumors cannot always be fit for the PDT of bacteria, especially Gram-negative bacteria (Malik et al., 1992). For instance, the cell wall of Gram-negative bacteria is thick and it has been a tough issue to deliver the photosensitizer into the Gram-negative bacteria. Hence, the Gram-negative bacteria are notoriously difficult to deal with (Malik et al., 1992).

**FIGURE 11:**
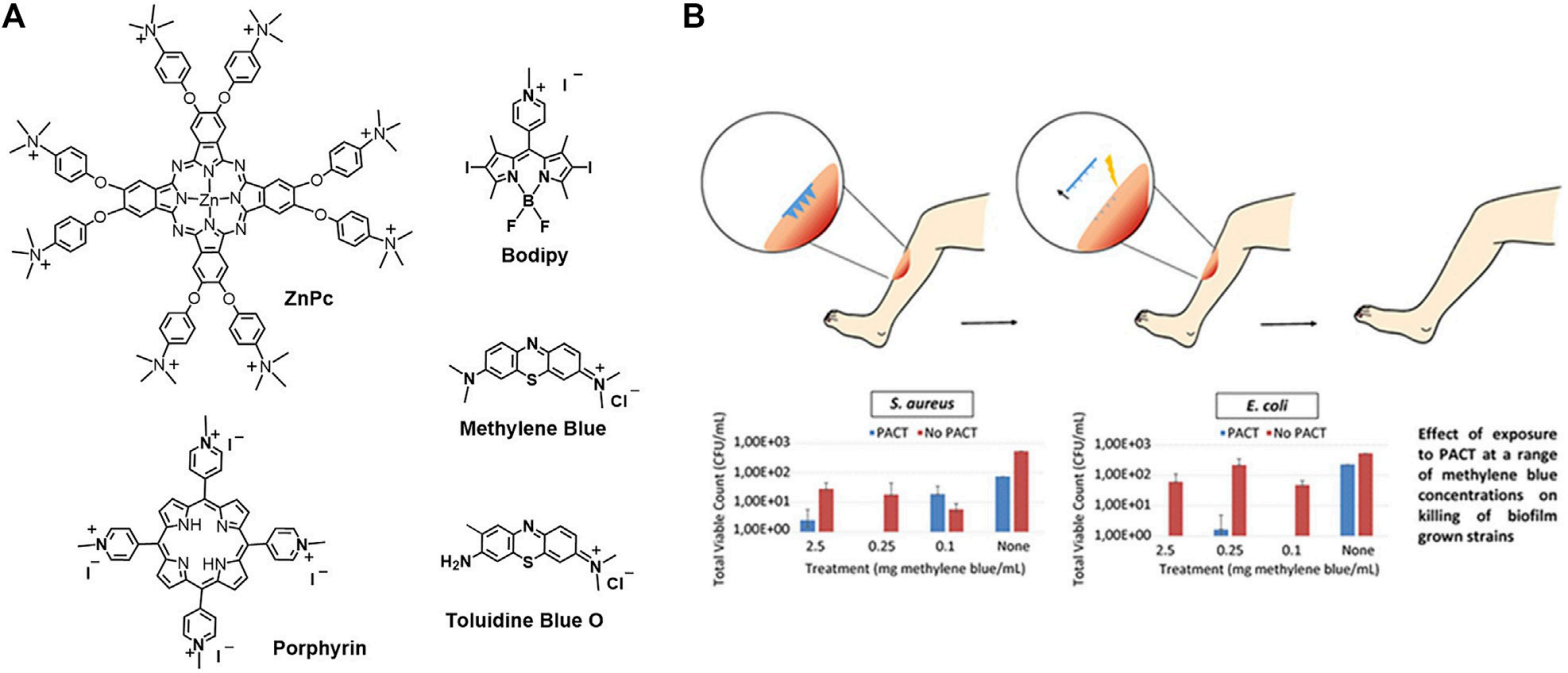
**(A)** Molecular structure of cationic photosensitizers; **(B)** methylene blue-loaded microneedle for photodynamic antimicrobial chemotherapy at a range of methylene blue concentrations on killing of biofilm grown strains *S. aureus* and *E. coli* (Caffarel-Salvador et al., 2015).

As the bacteria are heavily negatively charged, the cationic photosensitizers are beneficial for their penetration and targeting to bacteria *via* electrostatic interactions (Minnock et al., 1996; Chen et al., 2020). This is a kind of structure-inherent targeting. Cationic photosensitizers such as methylene blue, cationic porphyrins, cationic Bodipy, and cationic phenoxyphthalocyaninato zinc (II) are shown as examples in Figure 11A.

#### Active Targeting

Similar to the anti-tumor therapy, there is also active targeting strategy to achieve better PDT efficacy. As the structures of tumor cells and bacteria are different, the target point also varies. For instance, the anti-tumor therapy targets the tumor vasculature, tumor cells, and tumor subcellular organelles such as mitochondria, lysosome, nucleus, and endoplasmic reticulum. While the anti-bacterial therapy targets the bacteria membranes, bacterial exopolysaccharides (EPS), fimbriae, etc. For instance, various EPS are produced by bacteria to create a binding network between pathogens. Hence, bacterial EPS extracted from *Lactobacillus plantarum* can be utilized as targeting moiety (Li C. et al., 2018; Klausen et al., 2020). In addition, glycan is a commonly used targeting moiety with specific binding to lectins. Concerning to this aspect, different sugars such as mannose, sialic acid, and galactose have been utilized to target different pathogens. For instance, D-mannose has been used to bind to FimA protein of *Escherichia coli* (*E. coli*) (Krogfelt et al., 1990). As a deeper investigation and understanding to the bacteria, more and more target moieties are emerging.

#### Physical Penetration Targeting

As it has been introduced that the bacteria, especially Gram-negative bacteria, have thick cell walls, it makes them very difficult to deal with. Physical penetration by microneedles is an attractive strategy to go across the barriers and target the bacteria (Henry et al., 1998; Lee et al., 2008; Tuan-Mahmood et al., 2013; Donnelly et al., 2014; Kearney et al., 2014; Park et al., 2014). Methylene blue-loaded dissolving microneedles were constructed for antimicrobial PDT (Caffarel-Salvador et al., 2015). The microneedles were fabricated with aqueous blends of Gantrez® AN-139 co-polymer loading with different percentages of methylene blue. As presented in Figure 11B, the microneedles are in the micron range and are arranged in arrays. Loading high percentages of methylene blue (5% w/w) resulted in a decrease of needle height and microneedle strength. Loading suitable percentages of methylene blue (0.5% w/w) shows good mechanical strength that enables the insertion of the microneedles into the wound. The insertion distance is in the range of 378–504 μm, which is measured with a human tissue mimicker Parafilm™. The microneedles are expected to dissolve, followed by the release of the methylene blue. Upon photoirradiation, efficient bactericidal activity against *Staphylococcus aureus* (*S. aureus*) and *E. coli* biofilms was observed (Figure 11B).

### Bacterial-Activatable Polymeric Delivery System

Due to the encapsulation of EPS, the biofilm microenvironment is lack of oxygen, which leads to anaerobic glycolysis (Flemming et al., 2016). Recalling from the tumor-activatable polymeric delivery system section, the tumor tissues are also hypoxic. The anaerobic glycolysis or the anaerobic respiration contributes to the acidic and highly reductive microenvironment of both bacterial biofilms and tumor tissues (Gales et al., 2008; Chen et al., 2013; Koo et al., 2017). For instance, the pH in the methicillin resistant *Staphylococcus aureus* (MRSA) biofilm microenvironment is less than 5.5, and the concentration of GSH in *E. coli* biofilm is up to 10 mM (Fux et al., 2005; Gales et al., 2008; Chen et al., 2013; Klare et al., 2016). Hence, the microenvironment of bacteria is similar to that of tumor, which is hypoxic, acidic, highly reductive, and has abundant ROS. Taking advantage of the differences between bacteria and normal tissues, the activatable polymeric delivery system can be developed, achieving selectively killing of the bacteria while keeping the host normal tissue unaffected. Hence, decreased side effect is expected. Herein we presented some typical examples of activatable polymeric delivery systems for anti-bacteria PDT (Radovic-Moreno et al., 2012; Li F. et al., 2018; Zhao et al., 2019; Broenstrup et al., 2021; Wu et al., 2021; Ye et al., 2021).

#### Enzyme

**FIGURE 12:**
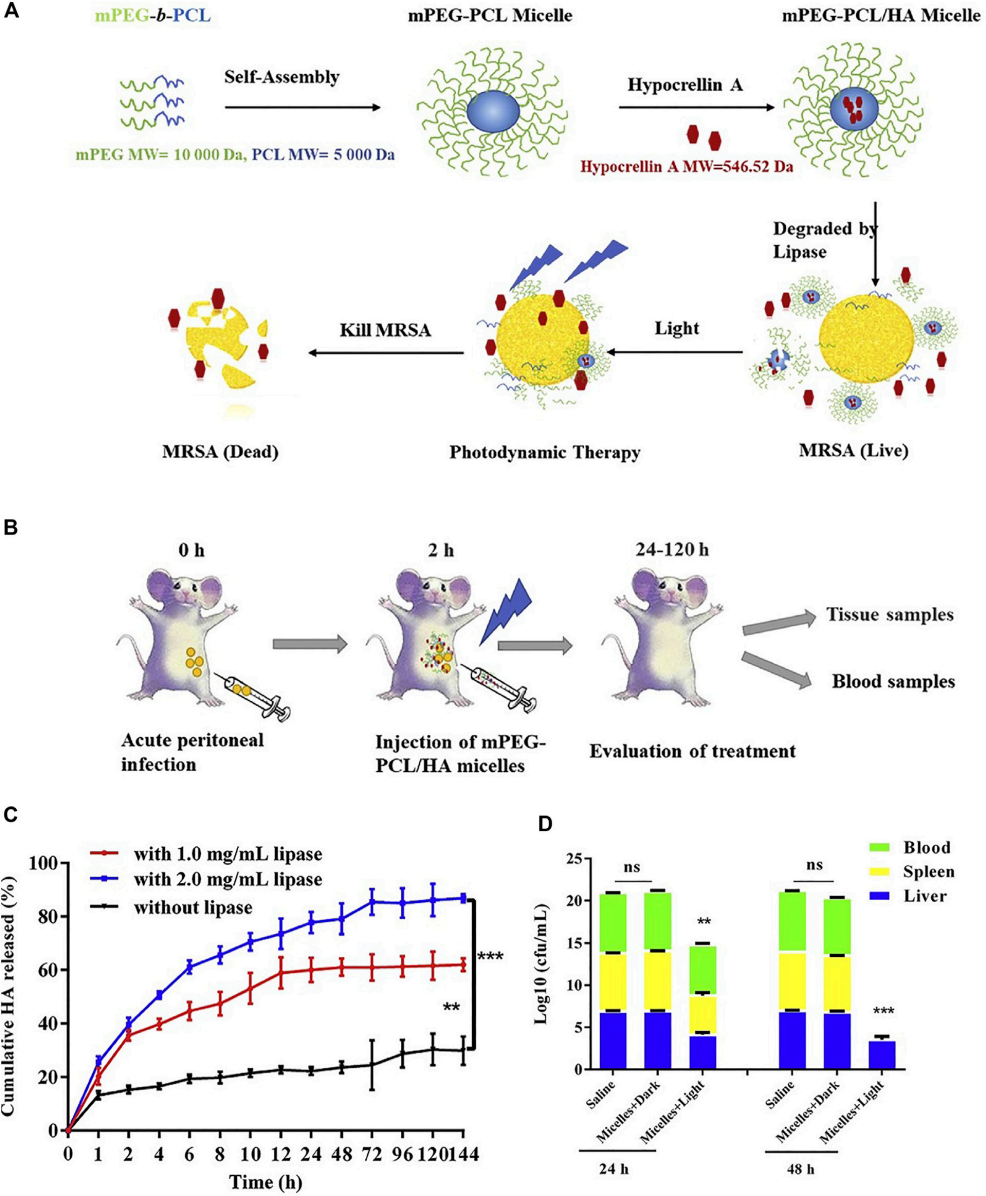
**(A)** Schematic illustration of the mPEG-PCL/HA micelles for enhanced photodynamic antibacterial activity; **(B)** schematic illustration of the mPEG-PCL/HA micelles for the *in vivo* treatment; **(C)** cumulative release of HA from micelles without or with lipase (1.0, 2.0 mg/ml); **(D)** total bacterial counts in the liver, spleen, and blood of mice after being treated with mPEG-PCL/HA micelles (HA dose: 5 mg/kg) in dark or light irradiation for 24 and 48 h. Reprinted with permission from Guo et al. (2020).

Hypocrellin A-loaded lipase sensitive polymer system was reported. The Hypocrellin A is a perylenequinoid pigment that is isolated from the tradition Chinese medicine and it is reported to have singlet oxygen production ability, hence it functioned as a photosensitizer. The lipase sensitive polymer methoxy poly (ethylene glycol)-blockpoly (ε-caprolactone) (mPEG-PCL) self-assembled to micelles, which is used to encapsulate the photosensitizer Hypocrellin A and enhance its water solubility. As shown in Figure 12A, this Hypocrellin A-loaded polymer micelles can be degraded by the bacterial lipase, along with the release of the photosensitizer. The release of Hypocrellin A in the presence of lipase in PBS was confirmed and presented in Figure 12C. An *in vitro* antibacterial study shows that the light irradiation significantly reduced the minimum inhibitory concentration (MIC) and minimum bactericidal concentration (MBC) values (0.69 and 1.38 mg/L, respectively), as compared with that in the dark (250 and 500 mg/L, respectively). This result demonstrated that the antibacterial activity is mainly attributed to PDT. An *in vivo* antibacterial study shows that the *MRSA* in spleen and blood is cleared up and the bacteria in liver is significantly reduced upon PDT (Figure 12D). Compared with the free Hypocrellin A, the polymeric micelles show slightly higher MIC and MBC values, which might be due to the incomplete release of Hypocrellin A. However, the lipase sensitive polymeric micelles achieve significantly increased survival rate, indicating that the lipase sensitive polymeric micelles are a potent polymeric system for combating MRSA infection.

#### pH

**FIGURE 13:**
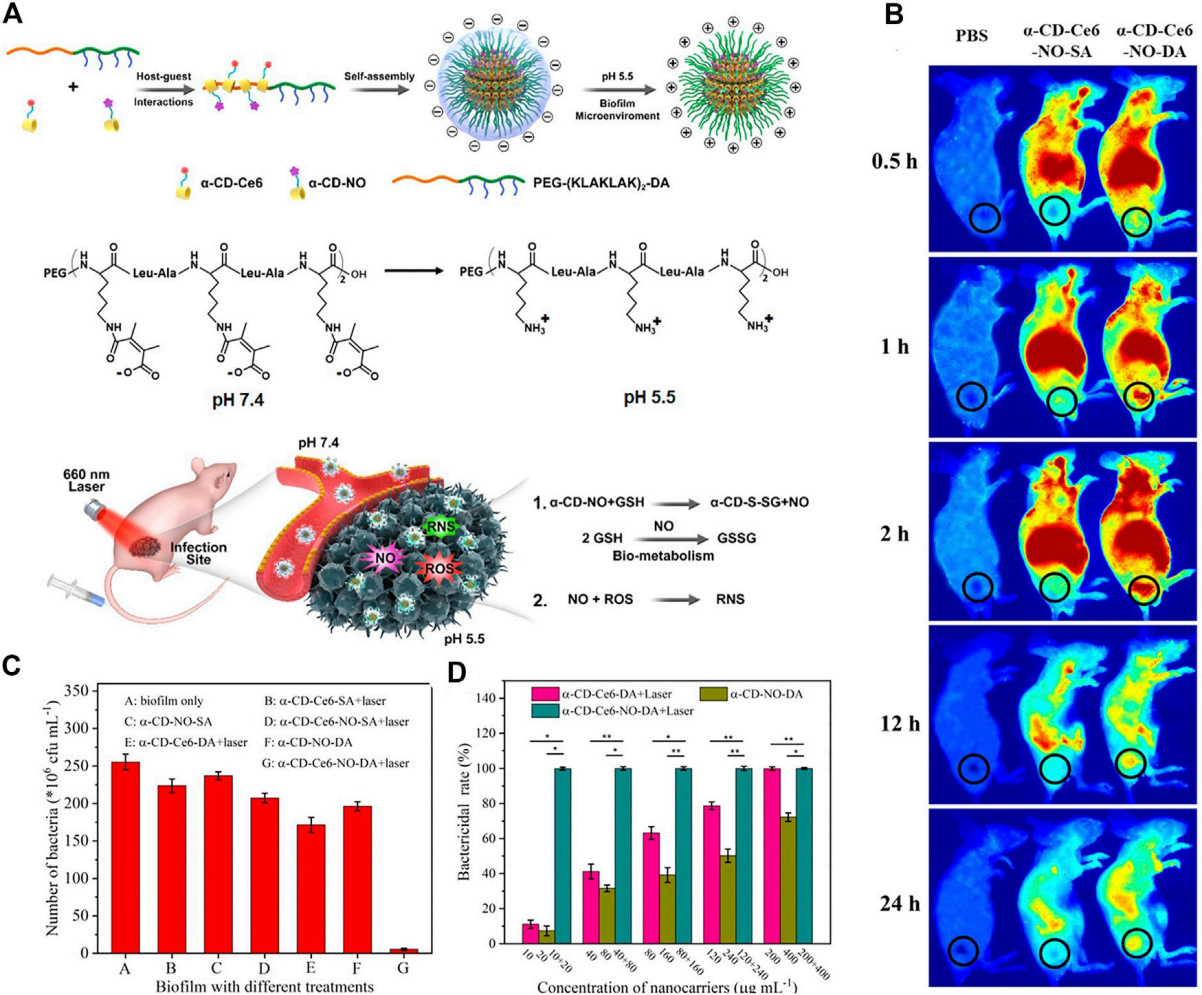
**(A)** Schematic diagram of the acid-activated charge reversal of PEG-(KLAKLAK)2-DA at pH 5.5 and the mechanisms of the MRSA biofilm associated infection eradication by synergistic effects between ROS and NO produced by α-CD-Ce6-NO-DA nanocarriers; **(B)** *in vivo* time-dependent body fluorescence imaging of the MRSA biofilm infected mice after various treatments; **(C)** related bactericidal results of different nanocarriers with the same Ce6 concentration under laser irradiation, characterized by the standard plate counting assay. **(D)** *In vivo* bactericidal rates of various treatments. Reprinted with permission from Hu et al. (2019).

A pH-sensitive, surface charge switchable supramolecular polymeric system was constructed (Hu et al., 2019). As shown in Figure 13A, the pH-sensitive poly (ethylene glycol) (PEG) block polypeptide copolymer [PEG-(KLAKLAK)2-DA] interacted with the α-CD prodrugs (PDT therapeutics α-CD-Ce6 and NO therapeutics α-CD-NO) *via* host-guest interaction, forming a negatively charged supramolecular nanocarrier at physiological pH. The negative charge is in favor of the long-term blood circulation. While in acidic conditions (pH = 5.5), the amide bond tends to be cleaved, leading to a switch of the surface charge of the nanocarrier. The obtained positive charge is beneficial for the penetration into biofilms due to the stronger interaction and adhesion to the negatively charged bacterial membrane. As compared with the surface charge unswitchable nanocarrier α-CD-Ce6-NO-SA, the surface charge switchable nanocarrier α-CD-Ce6-NO-DA shows enhanced penetration to biofilms and accumulation in MRSA biofilm infected area (Figure 13B). Hence, it is reasonable that upon light irradiation, enhanced bactericidal rate was observed for α-CD-Ce6-NO-DA as compared with α-CD-Ce6-NO-SA (Figure 13C and Figure 13D).

**FIGURE 14:**
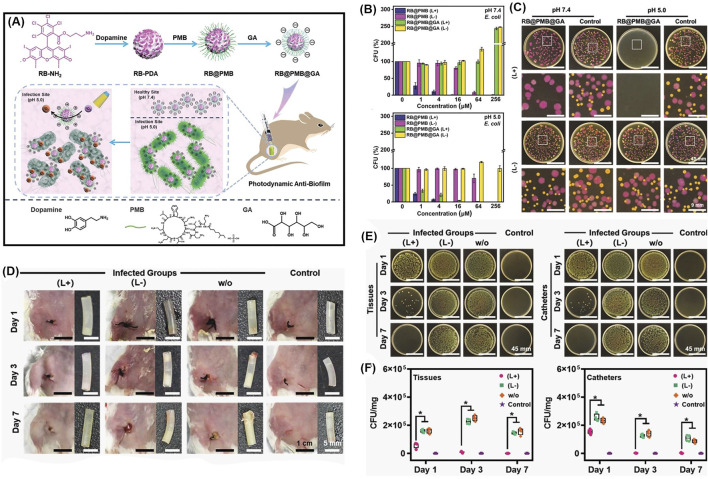
**(A)** Schematic illustration of the preparation process of photodynamic NPs for enhanced penetration and antibacterial efficiency in biofilms; **(B)** antibacterial activities against *E. coli* at pH 7.4 and pH 5.0, with or without irradiation; **(C)** photographs of *E. coli* labeled with the red protein and *S. aureus* colonies incubated together with the different treatments; **(D)** typical photographs of the incision areas and implanted catheters from mice under different treatments on Days 1, 3, and 7; **(E)** photographs of *P. aeruginosa* bacterial colonies, and **(F)** quantitative analysis of bacterial colony-forming units obtained from the tissues and catheters in each group. Reprinted with permission from Wu et al. (2021).

Another pH-sensitive charge reversible nanoparticle system was constructed for enhanced penetration and antibacterial PDT efficiency (Wu et al., 2021). As shown in Figure 14A, the photosensitizer Rose Bengal is dangled with dopamine, and then decorated with polymyxin B (PMB) and gluconic acid (GA) layer-by-layer. Resembling the isoelectric point of protein, the resulted nanoparticles (RB@PMB@GA) are negatively charged at physiological pH while they switched to positive charged when the pH is below 5.5, due to the presence of an amino group and a carboxyl group. Consequently, the antibacterial efficiency is greatly improved at pH = 5.0 (Figure 14B), due to the pH-sensitive charge inversion. To be specific, as presented in Figure 14C, only Gram-positive bacteria *S. aureus* were effectively killed upon light irradiation at pH = 7.4, while the Gram-negative bacteria *E. coli* were not affected unless in acidic condition (pH = 5.0). This indicates that the charge inversion in acidic condition enhanced the penetration and adhesion to bacteria. As the positively charged polymers are expected to quickly infiltrate biofilms *via* electrostatic interactions, the nanoparticles show outstanding biofilm penetration and eradication ability (Figures 14D–F).

#### ROS

**FIGURE 15:**
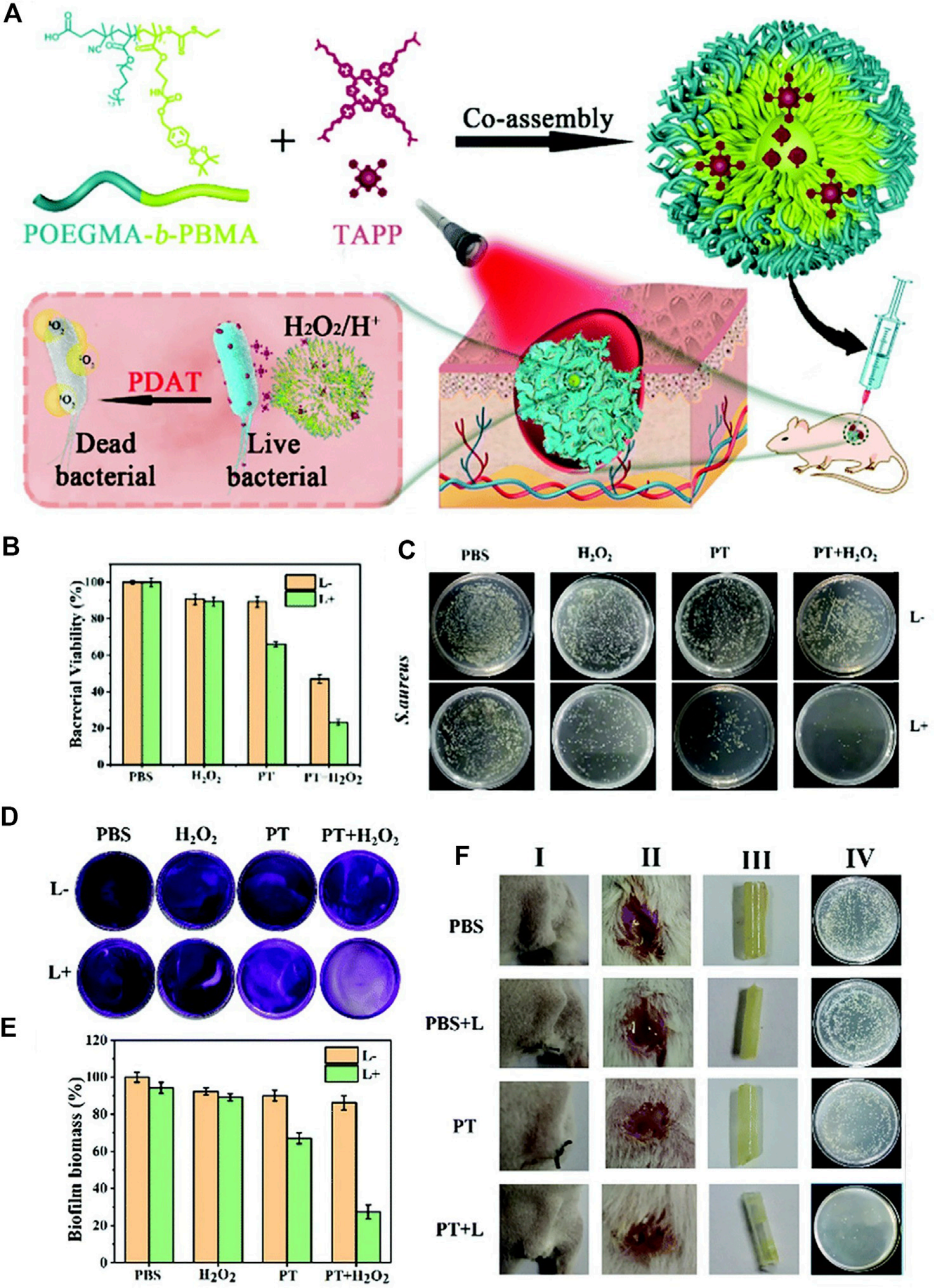
**(A)** Schematic diagram of a pH/H_2_O_2_ dual-responsive nanoplatform and its photodynamic antimicrobial therapy process; **(B)** bacterial viability of *S. aureus* treated with different conditions at pH = 5.5; **(C)** the corresponding *S. aureus* on agar plates after different treatments. Reprinted with permission from Zhao et al. (2021).

A pH/H_2_O_2_ dual responsive polymeric system to combat the *S. aureus* and its biofilm is presented in Figure 15A(Zhao et al., 2021) The polymer POEGMA-b-PBMA is assembled with a surface charge-switchable photosensitizer, 5,10,15,20-tetra-{4-[3-(N,N-dimethyl-ammonio) propoxy]phenyl} porphyrin (TAPP) into nanoparticles with an average diameter of 180 nm. The large amount of H_2_O_2_ in *S. aureus* reacts with the arylboronic ester moiety and induces the disintegration of the nanoparticles, followed by the release of TAPP. The TAPP is further protonated in an acidic bacterial microenvironment, which increases its hydrophilicity and reduces its self-quenching effect. As a result, enhanced fluorescence emission and singlet oxygen production ability of TAPP were observed in the presence of H_2_O_2_ at pH = 5.5. The *in vitro* antibacterial effect was examined (Figure 15B and Figure 15C). The zeta potential of the bacteria is increased from −10 to +2 mV when incubated with the nanoparticles, indicating the adherence ability of nanoparticles to bacteria. There was 80% of *S. aureus* killed by nanoparticles in the presence of H_2_O_2_ upon light irradiation (Figure 15B and Figure 15C). *In vivo* anti-biofilm activity of nanoparticles was quantified, and an obvious depression of biofilm was observed for the PDT treatment group.

#### GSH

Disulfide bond is frequently used as the GSH responsive linker to construct smart polymeric delivery systems for antibacterial PDT (Lu et al., 2018; Ye et al., 2020). For instance, the hyperbranched PEG was loaded with (Zinc)Porphyrins *via* disulfide and benzacetal linkers, which are sensitive to reductive (GSH) and acidic microenvironment of bacteria, respectively (Staegemann et al., 2018). The release of photosensitizer (Zinc)Porphyrins is confirmed *via* thin layer chromatography (TLC), size exclusion chromatography (SEC), dialysis and extraction, etc. The polymeric material shows significant phototoxicity against *S. aureus*. The benzacetal linker containing polymeric material was also applied for the PDT of tumor, which shows a less efficient depression of the tumor. This might be due to the more reductive and acidic microenvironment of bacteria than tumor, leading to more complete release of photosensitizers in bacteria and hence a better PDT efficacy. This result indicates that the treatments of cancer and bacteria are different.

## Summary and Outlook

Photodynamic therapy (PDT) has attracted great attention in the antitumor and antimicrobial area. This review introduces the smart polymeric delivery system for the PDT of tumor and bacterial infections. In particular, targeted and activatable polymeric delivery systems are highlighted. Due to the different structure of tumor and bacterial cells, the target point is different to some extent. For instance, the anti-tumor therapy targets the tumor vasculature, tumor cells, and tumor subcellular organelles such as mitochondria, lysosome, nucleus, and endoplasmic reticulum. While the anti-bacterial therapy targets the bacteria membranes, bacterial exopolysaccharides (EPS), and fimbriae, etc. On the other hand, the microenvironment of tumor and bacteria shares plenty of similarities such as hypoxic, acidic, highly reductive and have abundant ROS, etc. Hence, activatable polymeric delivery systems for both tumor and bacteria treatment have been developed. The activation is mainly *via* the following three strategies: (1) self-quenching and dequenching of photosensitizers due to aggregation and disintegration; (2) utilize another quencher to quench the triplet state of photosensitizer and dequenching upon cleavage of sensitive bonds; and (3) the change of size and surface charge to induce an enhanced internalization and penetration of tumor cells or bacterial cells.

Despite of the vigorous development of smart polymeric delivery system for PDT of tumor and bacteria, there are still some problems. First, there is great concern to the uncertainty of the molecular structure of polymers, which leads to the problem of reproducibility. Without a stable production and stable property, it is difficult for polymers to be applied in the clinic.

Second, concerning the passive targeting, recently the EPR effect is becoming a controversial topic and the general applicability of this EPR effect is challenged and still an open question.

Third, concerning the activatable polymeric delivery system, although the microenvironment of tumor/bacteria is different from that of normal tissues, there are still risks of inefficient specificity. For instance, ROS is not only abundant in tumor/bacteria cells, but also active in inflamed tissues. In addition, normal cells also have acidic subcellular compartments such as endosome and lysosome that are similar to those of cancer cells, which limits the pH-activatable theranostics to some extent.

Last but not the least, the reported polymeric delivery systems are usually complicated, and there is great concern to their toxicities. Developing non-toxic and simple polymeric delivery systems and forward their application from bench to bedside is highly desired.
